# Acupuncture improves anxiety and depression in patients with polycystic ovary syndrome: a systematic evaluation and meta-analysis

**DOI:** 10.3389/fmed.2026.1738629

**Published:** 2026-01-21

**Authors:** Rongzhen Ye, Yujia Sun, Han Yang, Jia Peng, Qingyun Tian, Songheng He, Siran Yao, Yefang Liu, Yu Liu, Jiao Chen

**Affiliations:** 1 School of Acupuncture and Tuina, Chengdu University of Traditional Chinese Medicine, Chengdu, China; 2 Chengdu Pidu District Hospital of Traditional Chinese Medicine, Chengdu, China; 3 The Third Affiliated Hospital of Chengdu University of Traditional Chinese Medicine (West District), Chengdu, China; 4 Division of Internal Medicine, Institute of Integrated Traditional Chinese and Western Medicine, West China Hospital, Sichuan University, Chengdu, China

**Keywords:** acupuncture, anxiety, depression, meta-analysis, polycystic ovary syndrome, randomized controlled trial

## Abstract

**Background:**

Acupuncture is increasingly utilized to address anxiety and depression in polycystic ovary syndrome (PCOS), yet evidence for non-pharmacological interventions remains limited. This study aimed to rigorously evaluate the efficacy and safety of acupuncture in alleviating anxiety and depression among women with PCOS, while exploring its potential mechanisms.

**Methods:**

Eight Chinese/English databases (CNKI, Web of Science, PubMed, Embase, etc.) were searched from inception to March 1, 2025. Two investigators independently screened studies, extracted data, and assessed quality via the Cochrane risk-of-bias tool. The meta-analyses were performed with RevMan 5.4. Additionally, data mining methods were used, including frequency statistics to analyze the frequency of acupuncture points and the meridians involved.

**Results:**

Twelve RCTs (*n* = 2,127 patients; acupuncture = 1,059, control = 1,068) were included. Compared with the control, acupuncture significantly reduced anxiety scores [MD = −6.42, 95% CI (−8.91, −3.56); *p* < 0.00001] and depression scores [MD = −5.89, 95% CI (−9.01, −2.78); *p* = 0.0002] versus controls. Acupuncture also improved testosterone [MD = −0.05, 95% CI (−0.11, 0.00); *p* = 0.05], BMI [MD = −0.70, 95% CI (−1.19, −0.21); *p* = 0.005], and the waist-hip ratio [MD = −0.06, 95% CI (−0.11, −0.01); *p* = 0.03], with no significant adverse effects [OR = 0.08, 95% CI (0.01, 0.81); *p* = 0.03]. The effects on insulin resistance were not significant [MD = −0.41, 95% CI (−1.18, 0.37); *p* = 0.31]. Data mining revealed that Foot Taiyin Spleen Meridian (SP), Conception Vessel (CV), and Foot Yangming Stomach Meridian (ST) were the most frequently used, and the most commonly used combination of points included SP6, LR3, and ST36.

**Conclusion:**

Acupuncture, particularly manual and short-term protocols, is a safe and effective adjunct for reducing anxiety and depression in PCOS. These benefits may be mediated via modulation of androgen levels, adiposity, and neuroendocrine pathways. Nevertheless, conclusions are limited by sample size, methodological heterogeneity, and inadequate adverse event reporting. Higher-quality RCTs are needed to confirm the safety and efficacy of these methods.

**Systematic review registration:**

https://www.crd.york.ac.uk/PROSPERO/view/CRD420251000646, Identifier CRD420251000646.

## 1 Introduction

Polycystic ovary syndrome (PCOS) is an endocrine disorder characterized by clinical and/or biochemical hyperandrogenism, ovulatory dysfunction, and/or polycystic ovarian morphology, that predominantly affects reproductive-aged women (1). With a global prevalence of 2–26% (2), the incidence of PCOS increased by 54.3% between 1990 and 2019 (3). In China, it affects 5.61% of reproductive-aged women with increasing trends, constituting a major healthcare challenge (4). Notably, PCOS patients face significantly elevated risks of psychological comorbidities (5); the prevalence of anxiety and depression in this population is estimated to reach 22 and 30%, respectively (6), potentially linked to insulin resistance (IR) and hyperandrogenaemia (7). Hormonal dysregulation and concomitant symptoms (e.g., obesity, infertility, acne, hirsutism, and androgenetic alopecia) may trigger or exacerbate mood disorders (8). These psychological burdens substantially impair quality of life and pose societal concerns, underscoring the imperative to address mental health in PCOS management.

The 2023 International Evidence-Based Guideline for PCOS (9) points out that the etiology remains elusive with no curative treatment. For PCOS patients with comorbid moderate-to-severe anxiety/depression, psychotherapy or selected pharmacotherapy is recommended (9). However, conventional pharmacological treatments demonstrate limited efficacy and may carry the risk of toxic side effects such as gastrointestinal reactions and cardiovascular disease (10). Thus, investigating safe and effective alternative or complementary therapies is imperative.

Acupuncture, a cornerstone of complementary and alternative medicine (CAM), has been integrated into the management of neuropsychiatric conditions (11). Evidence indicates that it alleviates emotional symptoms in cancer, Parkinson’s disease, and gastrointestinal disorders by modulating neuroendocrine pathways and the gut-brain axis-mediated microbial balance (12–15). In addition, acupuncture may directly or indirectly ameliorate the anxiety-depression in individuals with PCOS via regulation of the neuropeptide Y, norepinephrine (NE), and serotonin (5-HT) systems (15, 16), suggesting that it is a potential therapy for psychological symptoms (17–19). While existing meta-analyses have focused on the endocrine effects of acupuncture in individuals with PCOS (20–22), evidence regarding improvements in mood disorders remains scarce. This study presents the first meta-analysis specifically evaluating the efficacy of acupuncture for anxiety and depression in individuals with PCOS while investigating the mechanistic links to insulin resistance, hyperandrogenaemia, and obesity, thereby providing evidence-based guidance for clinical practice.

## 2 Data and methods

This study adhered to the Preferred Reporting Items for Systematic Reviews and Meta-Analyses (PRISMA) guidelines and checklist (23, 24), with the protocol registered on PROSPERO (CRD420251000646).

### 2.1 Data sources and literature search strategy

Eight databases (CNKI, Wanfang, VIP, Duxiu, Web of Science, PubMed, Embase, and Medline) were searched up to March 1, 2025. We obtained RCTs of the use of acupuncture to improve anxiety and depression in patients with polycystic ovary syndrome, using the following terms: acupuncture, acupuncture therapy, electroacupuncture, ear acupuncture, polycystic ovary syndrome, micropolycystic ovary, stein-leventhal syndrome, and randomized controlled trial. For detailed search strategies, please refer to Supplementary Table S1.

### 2.2 Inclusion and exclusion criteria

The inclusion criteria were as follows:

1. Study type: randomized controlled trials (RCTs) published in Chinese or English.
2. Participants: Patients definitively diagnosed with PCOS (meeting the 2003 Rotterdam criteria or 2011 Chinese Medical Association diagnostic standards), regardless of age or disease duration.
3. Interventions: Treatment group: Acupuncture alone (including body acupuncture, electroacupuncture, or auricular acupuncture) or acupuncture combined with conventional drug therapy. Control group: Sham acupuncture, waitlist/no treatment control, conventional drug therapy, or lifestyle interventions.
4. At least one of the following outcome measures: primary outcomes: anxiety status (assessed via the Self-Rating Anxiety Scale (SAS)) and depression status (assessed via the Self-Rating Depression Scale (SDS)). The secondary outcomes included testosterone (T), homeostatic model assessment for insulin resistance (HOMA-IR), body mass index (BMI), waist–hip ratio (WHR), and adverse reactions (e.g., bleeding, poor appetite, and abdominal pain).

Exclusion criteria:

1. Study type: Non-randomized studies, protocol papers, conference abstracts, case reports, review articles, editorials, or animal studies.
2. Participants: Studies focusing on syndromes other than PCOS (e.g., simple ovarian cysts, other endocrine disorders) or studies where PCOS patients constituted a minority of a mixed population without separable data.
3. Intervention and Control: Studies where the experimental intervention was not acupuncture or where acupuncture was a minor adjunct to another primary therapy (e.g., surgery, intensive psychotherapy). Studies where the control group received an active acupuncture treatment (e.g., different acupuncture protocol) rather than a credible control (sham, no treatment/blank, drug, or lifestyle). Studies where the type of control (e.g., sham vs. blank) could not be clearly determined from the report.
4. Outcomes: Studies that did not report at least one of the pre-specified primary outcomes (anxiety assessed by SAS or depression assessed by SDS).
5. Data and Reporting: Studies with missing, incomplete, or obviously erroneous key data (e.g., mean, standard deviation, sample size for outcomes) that could not be obtained or reasonably imputed after contacting the authors. Duplicate publications or secondary analyses of already included trials without new primary data.

### 2.3 Literature screening process

Two investigators (R.Z.Y. and Y.L.) independently (1) eliminated duplicate records via EndNote X9.1 software on the basis of eligibility criteria; (2) conducted preliminary screening by reviewing titles and abstracts to exclude nonconforming studies; and (3) performed full-text assessment for secondary screening to determine the final included trials. Key data, including researcher names, publication year, sample size, intervention protocols, outcome measures, and risk of bias assessments, were extracted into Excel spreadsheets. Disagreements during screening were resolved through consensus discussions or arbitration by a third researcher (J.C.).

### 2.4 Quality evaluation

The Cochrane risk of bias tool was used to evaluate potential biases in the included studies. The assessment domains included random sequence generation, allocation concealment, blinding of participants and personnel, blinding of outcome assessment, incomplete outcome data, selective outcome reporting, and other sources of bias.

The quality of evidence for each outcome was assessed by two independent researchers (R.Z.Y. and Y.L.) using the evaluation (GRADE) system, with discrepancies resolved through consultation with a third expert (J.C.). Within the GRADE framework, evidence was rated as “high”, “moderate”, “low”, or “very low” based on the following criteria: risk of bias, inconsistency, imprecision, indirectness, and publication bias. Specifically, inconsistency was judged based on the direction and magnitude of effect estimates, the overlap of confidence intervals, and the I^2^ statistic (where I^2^ > 50% indicated substantial heterogeneity that could downgrade the evidence). Publication bias was considered based on the results of funnel plot inspection and statistical tests as detailed in the Statistical Analysis section below. The detailed GRADE assessments are summarized in Supplementary Table S3.

### 2.5 Statistical analyses

RevMan 5.4.1 software was used for statistical analysis. All outcome data were continuous variables, expressed as the mean difference (MD) with 95% confidence interval (CI). Heterogeneity between studies was assessed via the I^2^ statistic: I^2^ ≤ 50% and *p* ≥ 0.05 indicated low heterogeneity, warranting a fixed-effects model; I^2^ > 50% or *p* < 0.05 indicated substantial heterogeneity, warranting a random-effects model. To explore potential sources of heterogeneity, pre-specified subgroup analyses were conducted based on acupuncture modality, treatment duration, and sample size. Sensitivity analysis was performed by sequentially removing each individual study to examine the stability of the pooled results, particularly for outcomes with high heterogeneity. Assessment of publication bias involved visual inspection of funnel plots for asymmetry. For outcomes that included 10 or more studies (i.e., anxiety and depression), Egger’s linear regression test was performed using Stata software 18.0 to quantitatively assess small-study effects. Additionally, acupuncture prescription patterns were analyzed using frequency statistics, and meridian–acupoint networks were visualized with Cytoscape software 3.9.0.

## 3 Results

### 3.1 Results of the literature search

**Figure 1:**
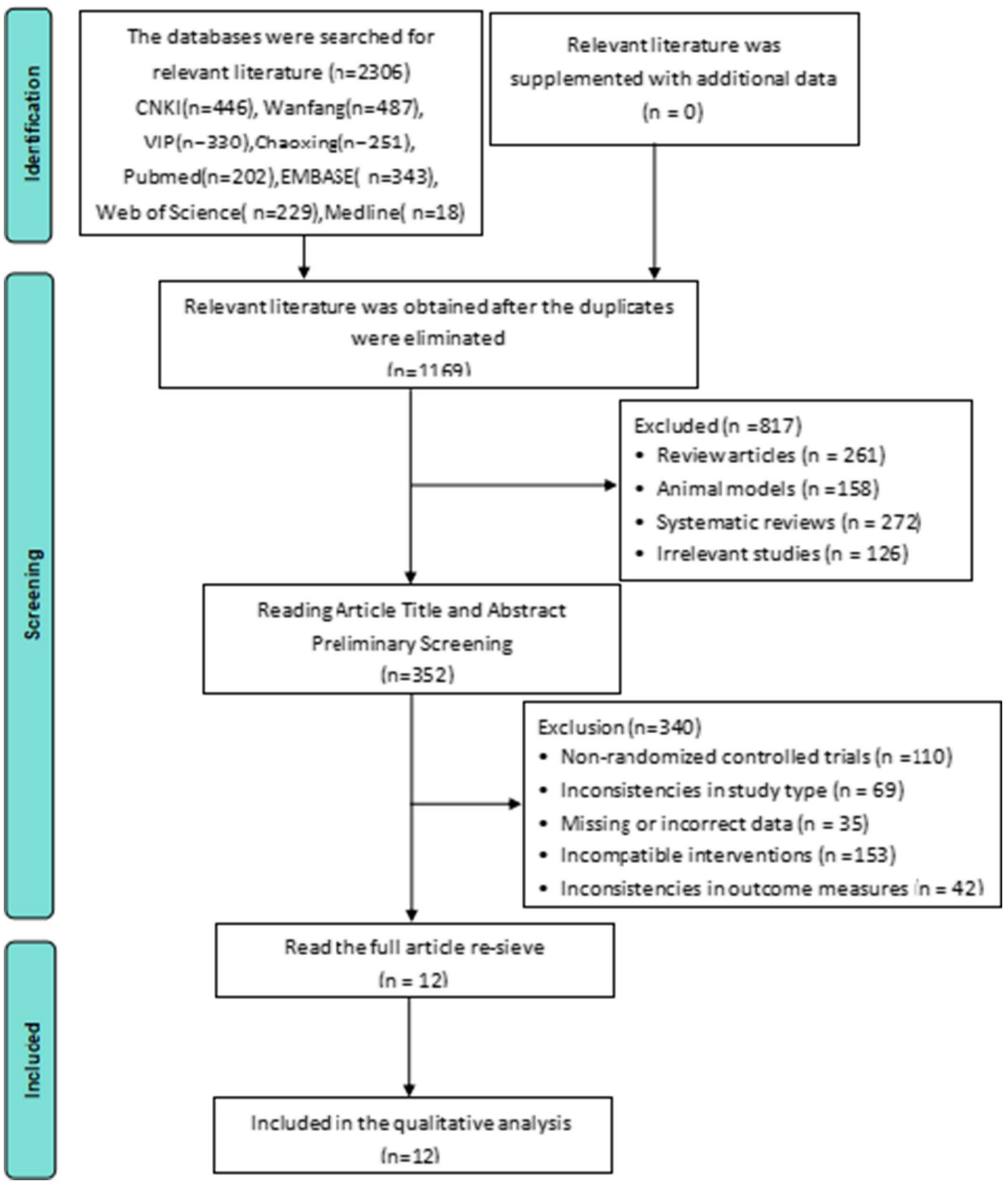
Literature screening process (PRISMA framework).

The initial search identified 2,306 records. After 1,137 duplicates were removed, 817 articles were excluded through title and abstract screening. The application of the inclusion/exclusion criteria resulted in the exclusion of 340 articles, yielding 12 RCTs (25–36) for final inclusion (Figure 1).

### 3.2 Basic characteristics of included studies

**Figure 2:**
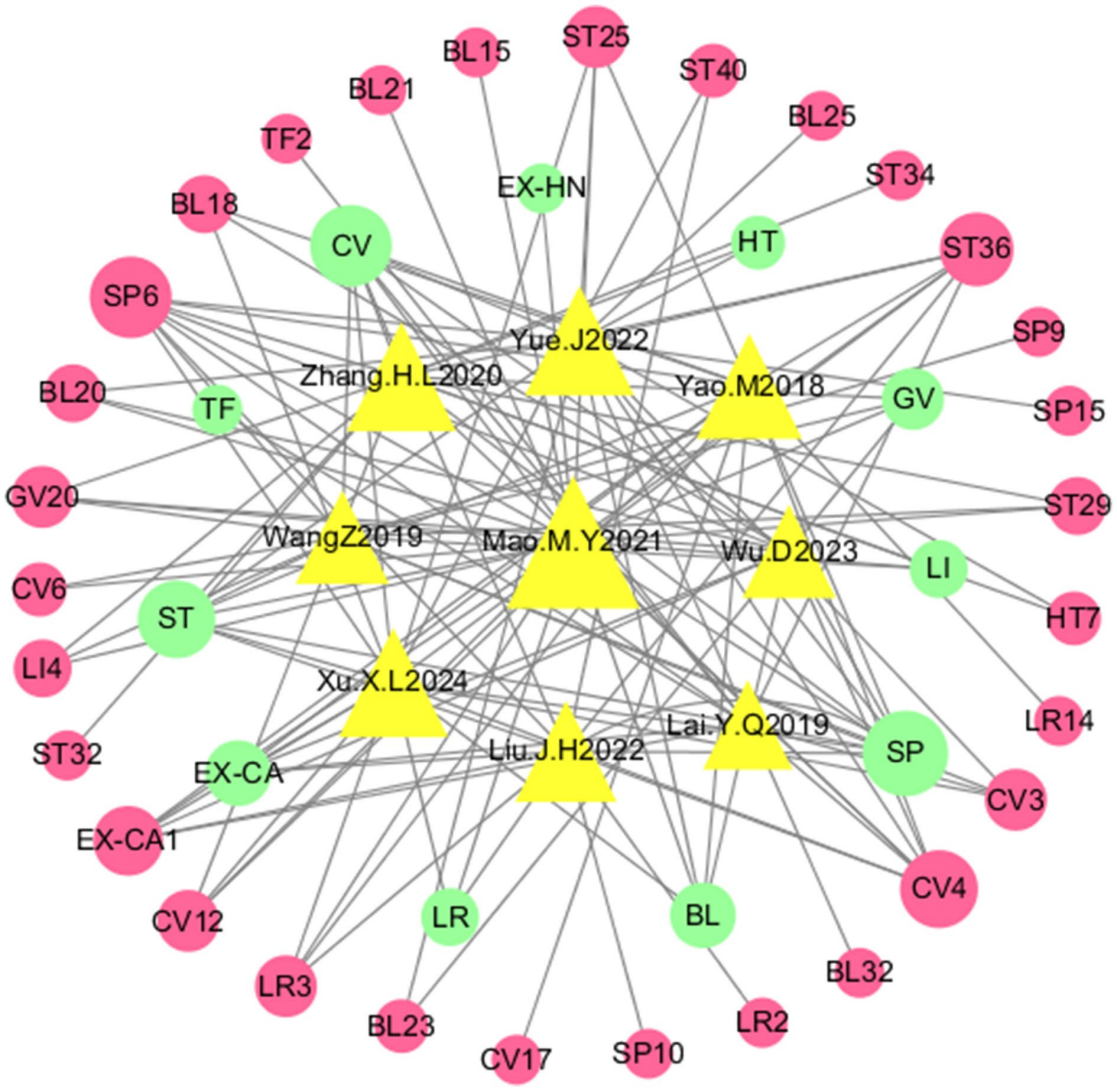
Network diagram of the meridian-acupoint usage frequency. SP6, Sanyinjiao; ST36, Foot Sanli; CV4, Guanyuan; LR3, Taichong; GV20, Baihui; EX-CA1, Uterus; ST25, Tianshu; CV3, Zhongji; BL20, Spleen Yu; BL18, Liver Yu; ST29, Guilai; LI4, Hegu; CV6, Qihai; CV12, Zhongkou; ST40, Fenglong; HT7, Shenmen; BL23, Kidney Yu; SP10, Sea of Blood; LR2, Xingma TF2, Ovary; EX-HN3, Indigo; BL21, Stomach; BL15, Heart; SP9, Yinlingquan; CV7, Tanzhong; LR14, Xiemen; SP15, Dahang; BL26, Guanyuan; BL32, Jiyu; ST32, Fubu; ST34, Liangqiu; SP, Spleen meridian; ST, Stomach meridian; CV, Ren meridian; BL, Bladder meridian; LR, Liver meridian; GV, Vessel meridian; LI, Large Intestine meridian; HT, Heart meridian; EX, Extra meridian points.

Twelve RCTs involving 2,127 patients (acupuncture group: *n* = 1,059; control group: *n* = 1,068) were included. The publication years ranged from 2013–2024, with 3 English-language studies and 9 Chinese-language studies. The outcome measures included SAS scores [12 studies (25–36)], SDS scores [10 studies (25, 27–32, 34–36)], testosterone (T) levels [5 studies (30, 32–35)], HOMA-IR values [5 studies (25, 28, 30, 33, 36)], BMI values [7 studies (25, 28, 30, 33–36)], WHR [4 studies (25, 28, 30, 33)], and adverse events [4 studies (30, 32, 33, 36)]. Interventions primarily consisted of manual acupuncture (27, 29, 31, 32, 34, 36) and electroacupuncture (25, 26, 28, 30, 33, 35). The treatment duration spanned 3–6 menstrual cycles, with 30-min sessions administered 2–3 times weekly. The detailed characteristics are presented in Tables 1, 2 and Figure 2.

**Table 1:** Characteristics of included studies.

Year of researcher	Country	Sample size (*n*)	Mean age (years)	Intervention		Course treatment (menstrual cycle)	Outcome indicators	Adverse events
		T/C	T/C	T/C	T/C			
Chang H. 2023 (25)	China	458/468	27.97 ± 3.33/27.97 ± 3.33	EA	EA	4	①②④⑤⑥	Not reported
Lai Y. Q. 2019 (26)	China	30/30	27.91 ± 4.41/28.36 ± 4.52	EA + TCM	CC + TCM	3	①	Not reported
Li J. H. 2022 (27)	China	45/45	31.02 ± 3.31/30.87 ± 3.89	MA + TCM + C	CC + HMG	3	①②	Not reported
Mao M. Y. 2021 (28)	China	54/54	-	EA + CPA/EE	EA + CPA/EE	3	①②④⑤⑥	Not reported
Wang Z. 2019 (29)	China	27/27	-	MA	SA	2	①②	Not reported
Wu X. K. 2017 (30)	China	250/249	28.20 ± 3.40/27.80 ± 3.40	EA + CC	SA + CC	4	①②③④⑤⑥	T: 34 cases; C: 16 cases
Wu D. 2023 (31)	China	30/30	21.89 ± 9.71/22.33 ± 9.49	MA + LET	LET	3	①②	Not reported
Xu X. L. 2024 (32)	China	35/35	29.39 ± 4.54/28.79 ± 4.67	MA + TCM	CPA/EE	3	①②③	T: no adverse reactions; C: 1 case of nausea
Yao M. 2018 (33)	China	50/50	27.8 ± 4.8/28.2 ± 4.5	EA	MET	6	①③④⑤⑥	T: 1 case of bleeding at the acupuncture point after the needle was discharged; C: 2 cases of poor appetite, 7 cases of gastrointestinal reaction, 1 case of headache and 1 case of skin rash.
Yue J. 2022 (34)	China	30/30	27 ± 5/27 ± 5	MA	SA	3	①②③⑤	Not reported
Zhang H. L. 2020 (35)	China	20/20	29 ± 2/28 ± 3	EA + E	E	4	①②③⑤	Not reported
Zhang S. K. 2024 (36)	China	30/30	28.99 ± 3.58/29.59 ± 3.76	MA + CPA/EE	CPA/EE	3	①②④⑤	T: 3 cases of bleeding at the acupoints after the needles were discharged; C: no adverse reactions occurred

T, Treatment group; C, Control group; MA, Manual acupuncture; EA, Electroacupuncture; SA, Sam acupuncture; TCM, Traditional Chinese Medicine cycle therapy/herbal tonics; E, Lifestyle management; CC, Clomiphene; MET, Metformin hydrochloride; CPA/EE, Cyproterone ethinylestradiol tablets; LET, Letrozole; HMG, Urotensin; WM, Western medical therapy. Outcome indicators: ① Anxiety status; ② Depression status; ③ Testosterone T; ④ Insulin resistance index HOMA-IR; ⑤ Body mass index BMI; ⑥ Waist-hip ratio WHR.

**Table 2:** Acupuncture regimens were included in the literature.

Year of researcher	Acupuncture points (treatment group)	Acupuncture method	Treatment duration
Chang H. 2023 (25)	Not reported	EA	RNS 30 min, Tow
Lai Y. Q. 2019 (26)	Zhongji CV3, Guanyuan CV4, Sanyinjiao SP6, Ashigaru ST36, Ovary TF2, Uterus EX-CA1	EA	RNS 20 min, Qd
Li J. H. 2022 (27)	Taichong LR3, Xingma LR2, Sanyinjiao SP6, Foot Sanli ST36, Sea of Blood SP10, Guanyuan CV4, Uterus EX-CA1, Fenglong ST40	MA	RNS 3 day, thw
Mao M. Y. 2021 (28)	Baihui GV20, Yintang EX-HN3, Spleen YU BL20, Stomach YU BL21, Heart YU BL15, Tianshu ST25, Guanyuan CV4, Qihai CV6, Guilai ST29, Zhongkou CV12, Hegu LI4, Taichong LR3, Adachi Sanli ST36, Sanyinjiao SP6	EA	RNS 30 min, Qod
Wang Z. 2019 (29)	Baihui GV20, Zhongji CV3, Qihai CV6, Guilai ST29, Sanyinjiao SP6, Hegu LI4, Yinlingquan SP9	MA	RNS 30 min, Tow
Wu X. K. 2017 (30)	Not reported	EA	RNS 30 min, Tow
Wu D. 2023 (31)	Baihui GV20, Guanyuan CV4, Ren Yu BL23, Liver BL18, Spleen BL20, Uterus EX-CA1, Sanyinjiao SP6	MA	RNS 30 min, Qd, 14 consecutive days per month
Xu X. L. 2024 (32)	Guanyuan CV4, Zhongji CV3, Uterus EX-CA1, Sanyinjiao SP6, Tianshu ST25, Zhongkou CV12, Ashansanli ST36, Fenglong ST40, Liver BL18, Taichong LR3	MA	RNS 3 day, Qod, Tow
Yao M. 2018 (33)	Tanzhong CV7, Liver Yu BL18, Tianshu ST25, Uterus EX-CA1, Ashigang Sanli ST36, Ximen LR14, Zhongkou CV12, Guanyuan CV4, Sanyinjiao SP6, Taichong LR3	EA	RNS 30 min, Thw
Yue J. 2022 (34)	Supine position group: Guanyuan CV4, Zhongji CV3, Tianshu ST25, Daxiang SP15, Shenmen HT7 (left), Shusanli ST36, Sanyinjiao SP6 Prone group: spleen yu BL20, kidney yu BL23, guanyuan yu BL26, ji-siao BL32	MA	RNS 30 min, Qd
Zhang H. L. 2020 (35)	Baihui GV20, Zhongguancun CV12, Guanyuan CV4, Guilai ST29, Fubu ST32, Liangqiu ST34, Sanyinjiao SP6, Shusanli ST36, Shenmen HT7, Hegu LI4	EA	RNS 30 min, Qod, Thw
Zhang S. K. 2024 (36)	Auricular points (specific not reported)	MA	RNS 30 min, Thw

MA, Manual acupuncture; EA, Electroacupuncture; RNS, Indwelling needle set; Qd, once a day; Qod, Once every other day; Tow, Twice weekly; Thw, three times weekly.

### 3.3 Quality assessment of literature

**Figure 3:**
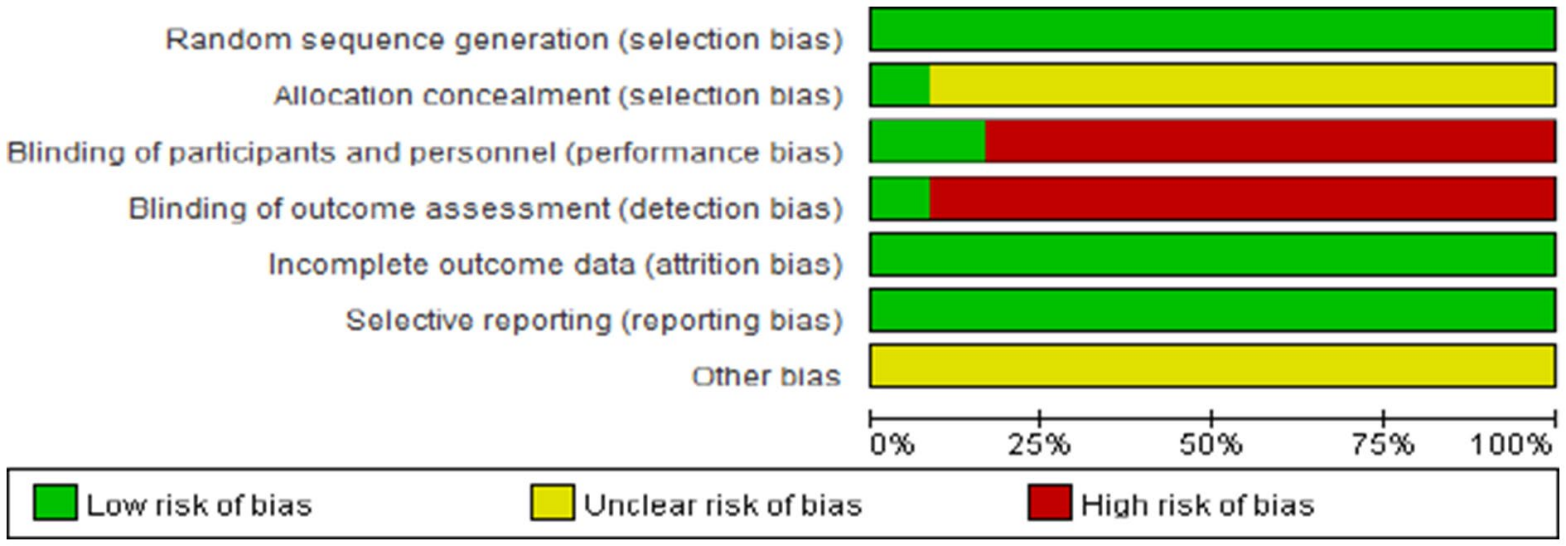
Risk of bias in the included studies.

**Figure 4:**
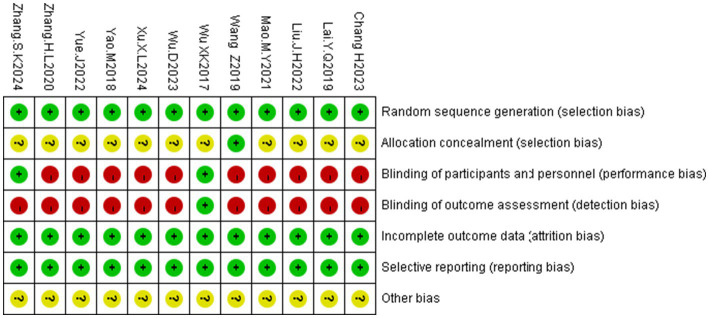
Risk bias summary plot of the included studies.

Ten RCTs (27–36) used random number tables for allocation, whereas two (25, 26) stated “randomization” without specifying methods. Three studies (29, 30, 36) implemented double- or single-blinding; allocation concealment was not reported in the remaining trials. All studies reported complete outcome data with no evidence of selective reporting. Other potential sources of bias were unclear. The risk of bias assessments are summarized in Figures 3, 4. According to GRADE (Supplementary Table S3), the quality of evidence ranged from very low to moderate.

### 3.4 Data mining analysis of meridian and acupoint utilization patterns

To systematically elucidate acupoint selection patterns in acupuncture treatment for PCOS-related anxiety and depression, we performed data mining and visualization analyses on acupuncture prescriptions extracted from the 12 included RCTs.

Frequency analysis (Supplementary Table S2) identified the Foot Taiyin Spleen Meridian (SP), Conception Vessel (CV), and Foot Yangming Stomach Meridian (ST) as the most frequently utilized meridians, collectively establishing the core meridian framework for acupuncture interventions in this context. At the acupoint level, the most frequently employed points were Sanyinjiao (SP6), Guanyuan (CV4), Zusanli (ST36), Taichong (LR3), and Zigong (EX-CA1), delineating a characteristic clinical acupoint combination profile.

The meridian-acupoint network diagram (Figure 2) constructed from these data further illustrated a selection pattern characterized by “a core structure comprising the Foot Yin Meridians, Conception Vessel, and Stomach Meridian, with specific acupoints functioning as central hubs.” Notably, Sanyinjiao (SP6)—the confluence point of the three Yin meridians of the foot (Spleen, Liver, and Kidney)—occupied a topologically central position within the network, exhibiting strong connections with Zusanli (ST36), Taichong (LR3), and Guanyuan (CV4).

#### 3.4.1 TCM theoretical interpretation of the core acupoint synergy

SP6, the confluence point of the three Yin meridians (Spleen, Liver, Kidney), is pivotal for regulating the Chong and Ren Vessels, nourishing Blood, and calming the Mind, thereby addressing the fundamental Yin deficiency and reproductive axis dysfunction in PCOS. LR3, the Source (Yuan) point of the Liver meridian, is the primary point for soothing Liver Qi stagnation, a key TCM pathogenesis for depression, irritability, and menstrual irregularities. ST36, the Sea (He) point of the Stomach meridian, strongly tonifies Qi and strengthens the Spleen, addressing the root of phlegm-dampness accumulation (manifested as obesity and metabolic dysfunction) and providing the material basis for physiological and emotional balance. The frequent co-occurrence of these points in the network diagram reflects a clinical strategy to simultaneously regulate the Liver (LR3), fortify the Spleen (ST36), and tonify the Kidneys and regulate the Chong-Ren (SP6), creating a holistic therapeutic approach to break the cycle of emotional distress, endocrine imbalance, and metabolic disturbance characteristic of PCOS (Supplementary Table S2).

#### 3.4.2 Interpretation of the network visualization

In the network diagram (Figure 2), circular nodes represent individual acupoints, with their size proportional to the frequency of use. Rectangular nodes represent meridians, color-coded for distinction. Solid lines connect acupoints to their parent meridian, while thicker lines between specific acupoints (e.g., connecting SP6, LR3, and ST36) indicate a higher frequency of co-occurrence within the same prescription, visually emphasizing their strong clinical association. This network topology not only confirms the quantitative findings from frequency analysis but also graphically underscores the principle of multi-point, multi-meridian synergy in TCM clinical practice for complex disorders like PCOS with psychological comorbidity.

In summary, this integrated data-driven and theory-informed analysis clarifies the preferred meridians and core acupoint combinations for acupuncture management of PCOS complicated by anxiety and depression. It demonstrates that contemporary clinical practice, as reflected in RCTs, aligns with classic TCM principles, providing an evidence-informed foundation for future clinical application and the development of standardized treatment protocols.

### 3.5 Meta-analysis results

#### 3.5.1 Anxiety state

**Figure 5:**
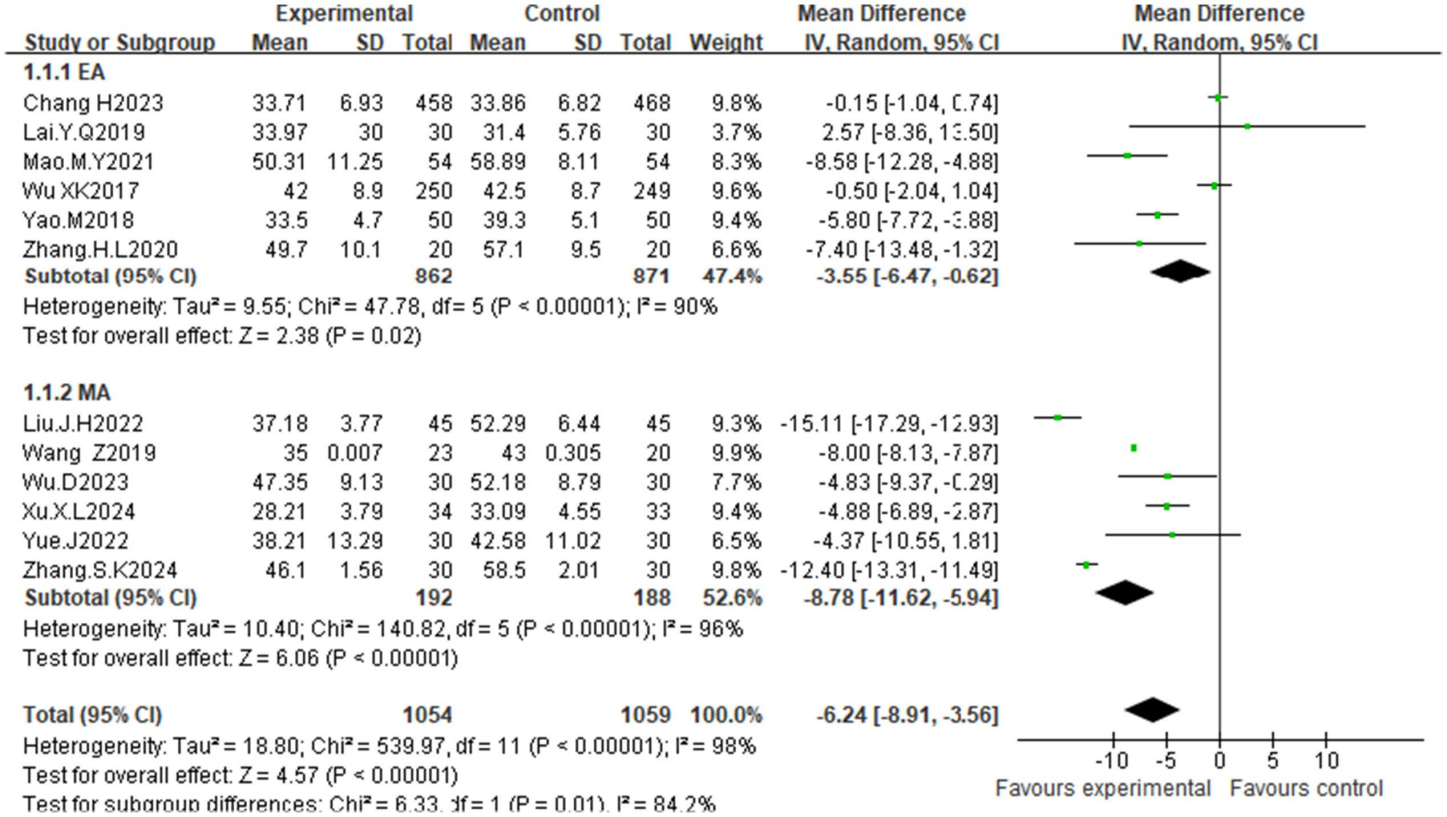
Forest plot of the effects of acupuncture on anxiety scores (subgroup analysis: EA vs. MA).

**Figure 6:**
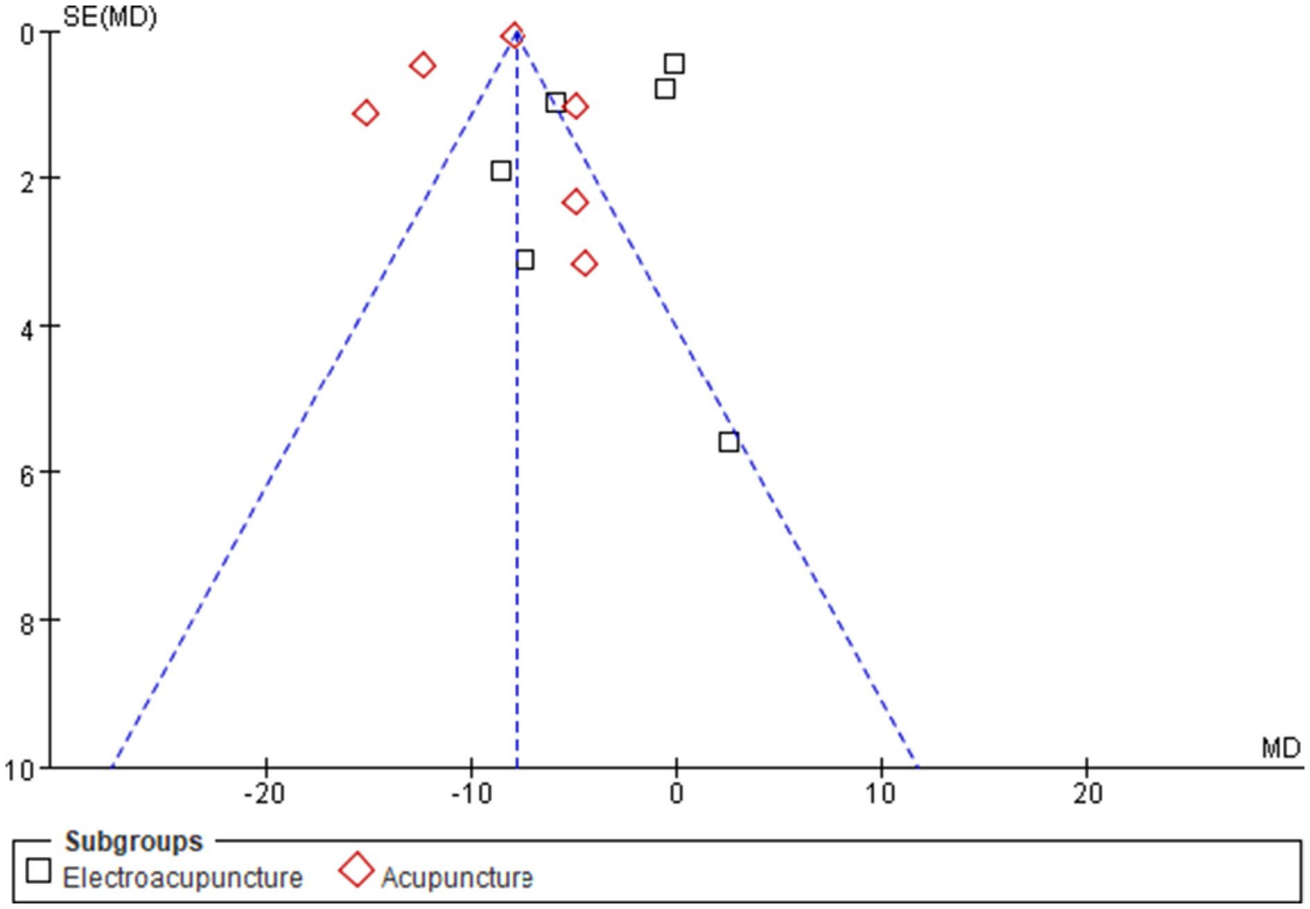
Funnel chart of anxiety states. Asymmetry was observed but Egger’s test was not significant (*p* = 0.445), possibly due to high heterogeneity.

Twelve studies (25–36) assessed anxiety in women with PCOS via the Self-Rating Anxiety Scale (SAS). Significant heterogeneity was observed (I^2^ = 98%, *p* < 0.00001), indicating the need for a random effects model. Meta-analysis demonstrated that acupuncture significantly reduced anxiety scores compared with those of controls [MD = −6.42, 95% CI (−8.91, −3.56); *p* < 0.00001]. Subgroup analyses by acupuncture modality, treatment duration, and sample size revealed reduced heterogeneity across all groups (Supplementary Table S1). Notably, manual acupuncture [MD = −8.78, 95% CI (−11.62, −5.94); *p* < 0.00001] showed significantly greater efficacy than electroacupuncture [MD = −3.55, 95% CI (−6.47, −0.62); *p* = 0.02], suggesting that treatment modality is a key source of effect heterogeneity (Figure 5). Funnel plot asymmetry indicated potential publication bias (Figure 6). Quantitative assessment via Egger’s test did not indicate significant publication bias (*p* = 0.445) (Supplementary Table S5). The observed funnel plot asymmetry is likely attributable to the high heterogeneity (I^2^ = 98%) among studies rather than systematic missing of negative trials.

#### 3.5.2 Depression states

**Figure 7:**
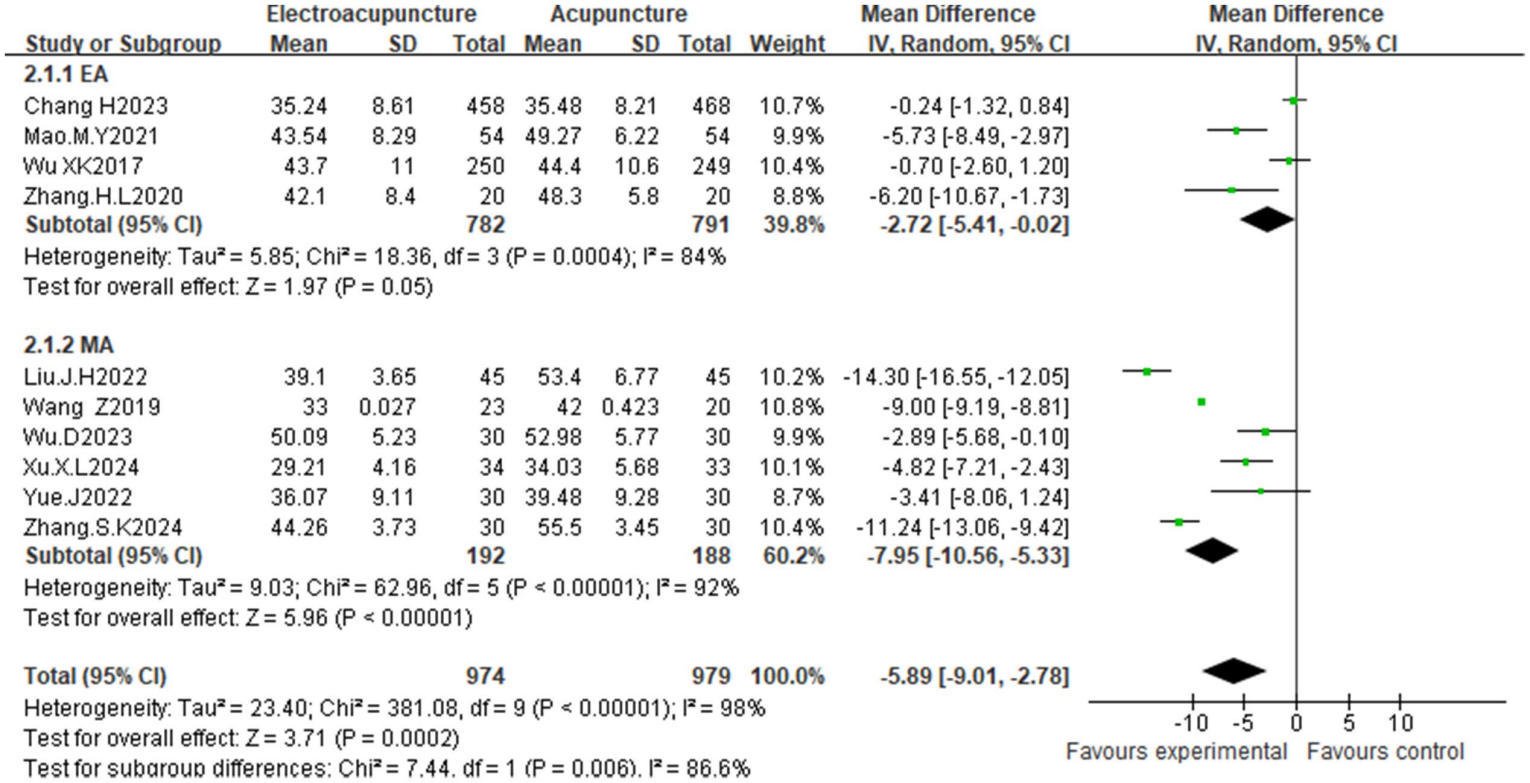
Forest plot of the effects of acupuncture on depression scores (subgroup analysis: EA vs. MA).

**Figure 8:**
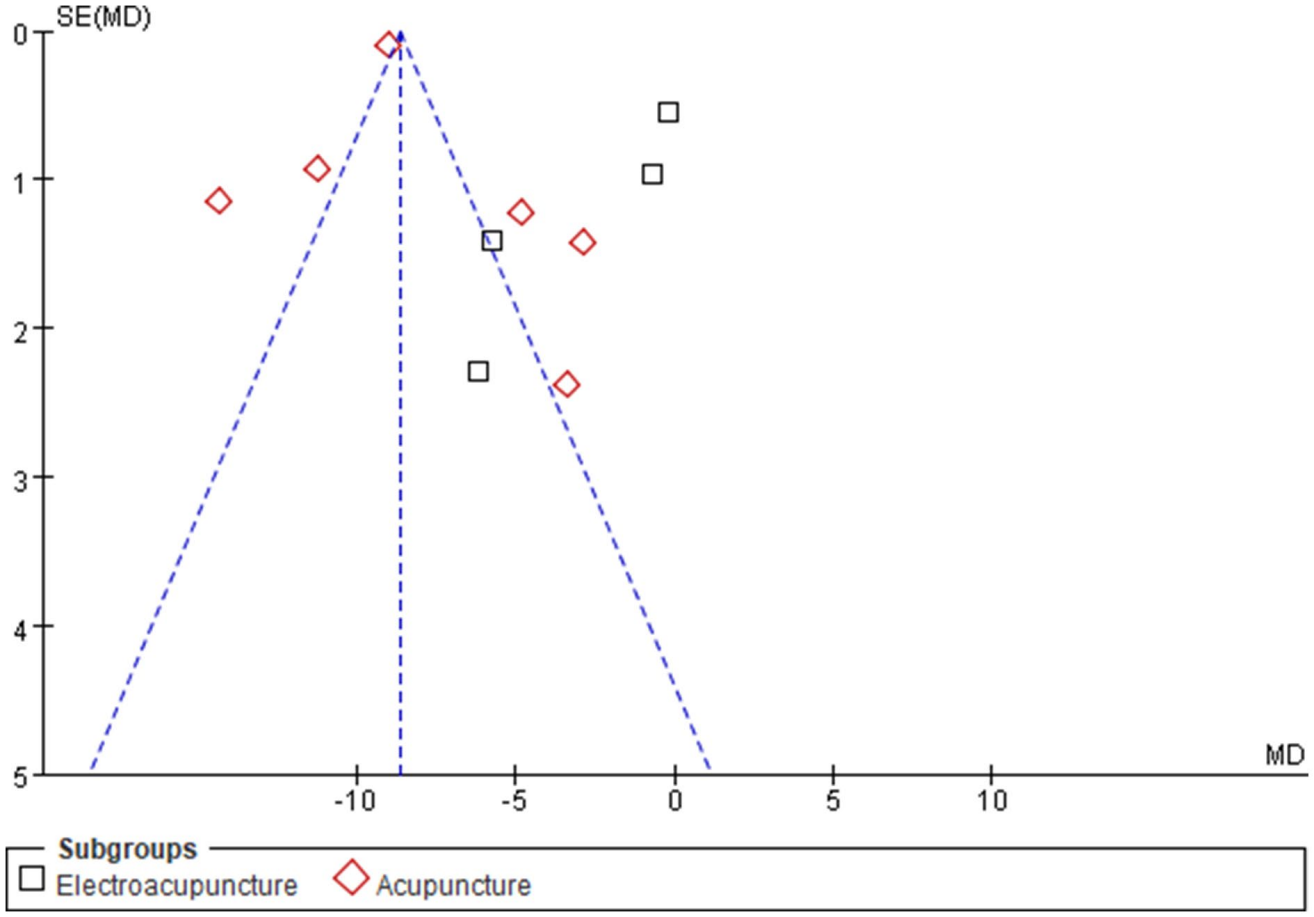
Funnel chart of depressive state. Asymmetry was noted; Egger’s test, however, was non-significant (*p* = 0.172).

Ten studies (25, 27–32, 34–36) evaluated depression via the Self-Rating Depression Scale (SDS). High heterogeneity (I^2^ = 98%, *p* < 0.00001) justified the use of a random effects model. Compared with the control condition, acupuncture significantly reduced depression scores [MD = −5.89, 95% CI (−9.01, −2.78); *p* = 0.0002]. Subgroup analyses (Supplementary Table S1) similarly revealed reduced heterogeneity, with manual acupuncture [MD = −7.95, 95% CI (−10.56, −5.33); *p* < 0.00001] demonstrating superior efficacy to electroacupuncture [MD = −2.72, 95% CI (−5.41, −0.02); *p* = 0.05], reinforcing modality as a determinant of treatment effects (Figure 7). The asymmetrical funnel plot distribution suggested a risk of publication bias (Figure 8). Similarly, Egger’s test for depression did not show statistical significance (*p* = 0.172) (Supplementary Table S5). The funnel plot asymmetry may thus reflect the substantial heterogeneity (I^2^ = 98%) across the limited number of included studies.

#### 3.5.3 Testosterone (T) levels

**Figure 9:**
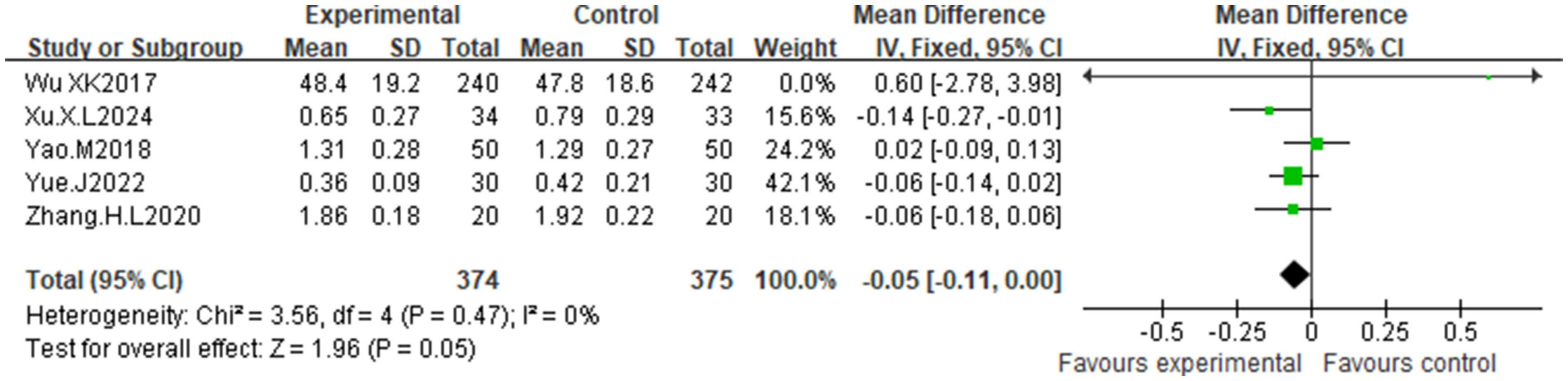
Effects of acupuncture on testosterone.

Five studies (30, 32–35) reported testosterone levels. Low heterogeneity (I^2^ = 0%, *p* = 0.47) justified the use of a fixed-effects model. Compared with the control, acupuncture significantly reduced testosterone levels [MD = −0.05, 95% CI (−0.11, 0.00); *p* = 0.05] (Figure 9).

#### 3.5.4 Homeostatic model assessment for insulin resistance

**Figure 10:**
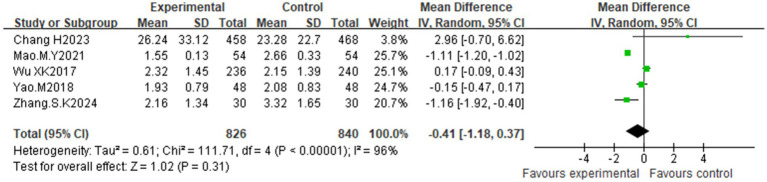
Effect of acupuncture on the insulin resistance index.

Five studies (25, 28, 30, 33, 36) assessed HOMA-IR. High heterogeneity (I^2^ = 96%, *p* < 0.00001) was detected via a random effects model. No statistically significant difference was observed between the acupuncture and control groups [MD = −0.41; 95% CI (−1.18, 0.37), *p* = 0.31] (Figure 10). Subgroup analyses (Supplementary Table S1) did not substantially reduce heterogeneity, indicating limited reliability of the results.

#### 3.5.5 Body mass index

**Figure 11:**
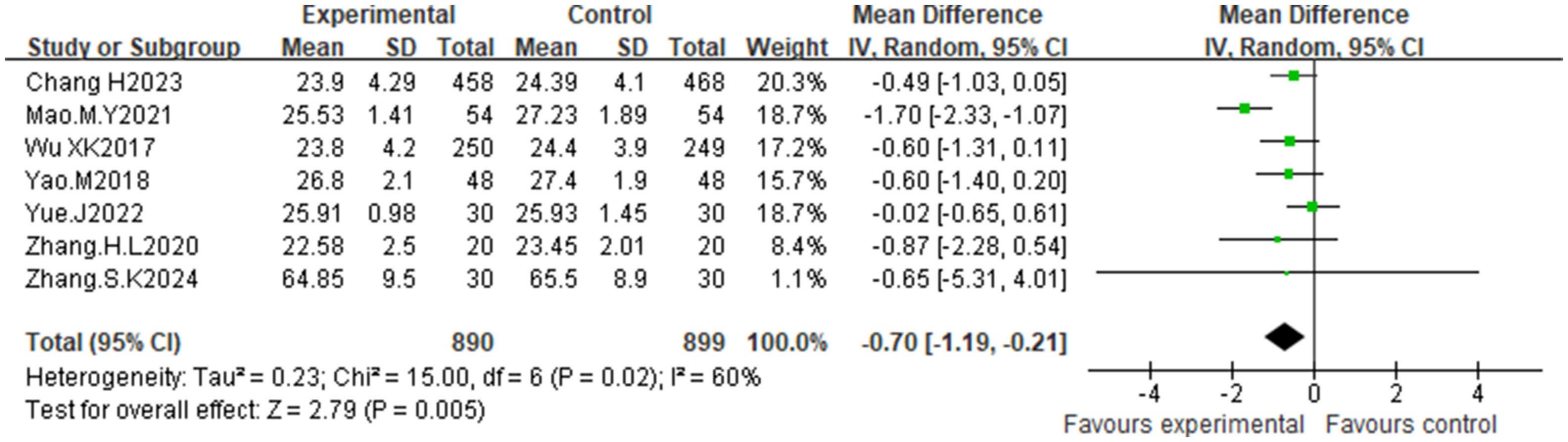
Effect of acupuncture on body mass index.

Seven studies (25, 28, 30, 33–36) reported BMI values. Moderate heterogeneity (I^2^ = 60%, *p* = 0.02) warranted a random effects model. Compared with the control diet, acupuncture significantly improved BMI [MD = −0.70, 95% CI (−1.19, −0.21); *p* = 0.005] (Figure 11). Subgroup analyses (Supplementary Table S1) revealed that the acupuncture modality and treatment duration were the primary sources of heterogeneity.

#### 3.5.6 Waist-to-hip ratio

**Figure 12:**
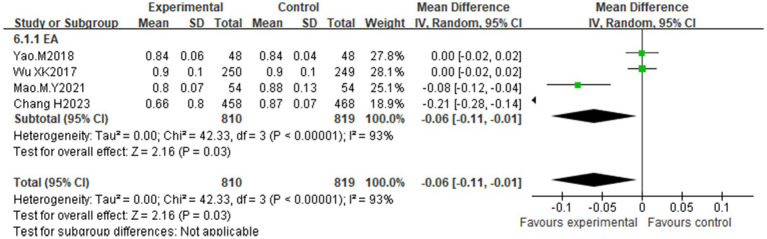
Effect of acupuncture on the waist-to-hip ratio.

Four studies (25, 28, 30, 33) evaluated WHR. High heterogeneity (I^2^ = 93%, *p* < 0.00001) necessitated a random effects model. Compared with the control, acupuncture significantly reduced the WHR [MD = −0.06, 95% CI (−0.11, −0.01); *p* = 0.03] (Figure 12). As all studies used electroacupuncture with large samples (*n* > 30), subgroup analysis was limited to treatment duration (Supplementary Table S1). Heterogeneity persisted, compromising the reliability of the results.

#### 3.5.7 Adverse events

**Figure 13:**
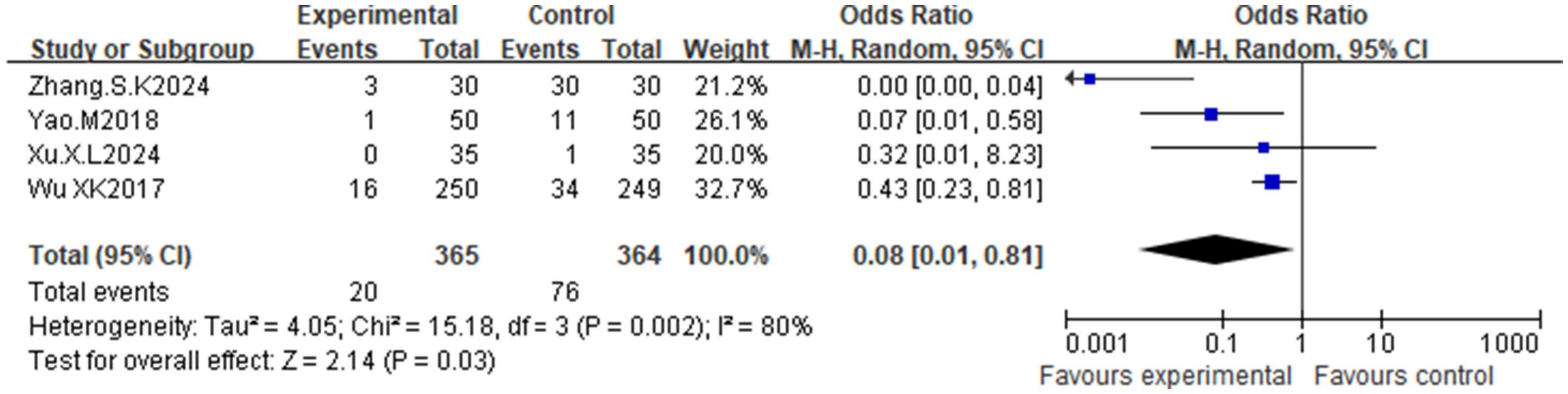
Effect of acupuncture on adverse reactions.

Four RCTs (30, 32, 33, 36) reported adverse events, primarily subcutaneous hemorrhage and gastrointestinal reactions (Table 3). The acupuncture group had a significantly lower incidence of adverse events [OR = 0.08, 95% CI (0.01, 0.81); *p* = 0.03] (Figure 13) with moderate heterogeneity (I^2^ = 81%, *p* = 0.002).

**Table 3:** Coverage of adverse reactions.

Year of researcher	Sample size (*n*) T/C	Intervention		Subcutaneous Hemorrhage	Gastrointestinal reaction			Other (rash, headache, dysmenorrhoea, etc.)	
		T	C	T	C	T	T	T	C
Wu X. K. 2017 (30)	250/249	EA + CC	SA + CC	1	14	3	9	12	11
Xu X. L. 2024 (32)	35/35	MA + TCM	CPA/EE	0	0	0	1	0	0
Yao M. 2018 (33)	50/50	EA	MET	1	0	0	9	0	2
Zhang S. K. 2024 (36)	30/30	MA + CPA/EE	CPA/EE	3	0	0	0	0	0

T, Treatment group; C, Control group; EA, Electroacupuncture; SA, Sham acupuncture; TCM, Traditional Chinese Medicine (TCM) cycle therapy/Chinese medicine (TCM) tonic; E, Lifestyle management; CC, Clomiphene; MET, Metformin hydrochloride; CPA/EE, Cyproterone ethinylestradiol.

### 3.6 Sensitivity analysis

Sensitivity analysis was conducted using SAS, SDS, HOMA-IR, and WHR as indicators. After sequentially excluding individual studies, the results indicated that the heterogeneity did not significantly decrease compared to the initial analysis. This suggests that the findings of this study are robust.

## 4 Discussion

### 4.1 Key findings and mechanistic insights

This systematic review of 12 RCTs evaluated the efficacy of acupuncture in alleviating anxiety and depression in women with PCOS while exploring the mechanistic links to insulin resistance (IR), hyperandrogenaemia, and obesity-related negative effects. Validated international scales (SAS/SDS) quantify subjective mood states, directly reflecting mental health status—a critical determinant of treatment adherence and quality of life in patients with PCOS. To investigate core pathological features, we analyzed key biomarkers, including testosterone (T), HOMA-IR, BMI, and the waist–hip ratio (WHR), elucidating the integrated mechanisms of acupuncture through endocrine axis modulation, metabolic improvement, and the regulation of energy balance/adipose tissue distribution. Most outcomes exhibited substantial heterogeneity (*I^2^* range: 0–100%; wide *Tau^2^* and *Chi^2^* distributions), indicating significant effect size variations. These discrepancies likely originated from inconsistencies in acupuncture modalities, treatment durations, and sample sizes. Subgroup analyses of outcome measures revealed reduced intergroup heterogeneity for most endpoints (Supplementary Table S4).

Collectively, acupuncture demonstrated superior efficacy in improving anxiety/depression scores, reducing testosterone levels, decreasing body weight, and optimizing adiposity distribution compared with controls—although not for HOMA-IR. Safety assessments, although limited by few adverse event reports, have consistently indicated a lower incidence and severity of acupuncture-related adverse events (primarily subcutaneous hemorrhage), supporting its favorable safety profile. Crucially, the risk of bias assessment necessitates caution in interpreting these findings because of potential publication bias in the included studies.

Women with PCOS exhibit elevated rates of depression (30–50%) and anxiety disorders (22–44%)—significantly higher than those in the general population, particularly among infertile patients (37, 38). Clinical symptoms overlap between depression and PCOS, with obesity, insulin resistance (IR), and hyperandrogenaemia constituting shared pathological underpinnings (39, 40). IR, present in 50–80% of PCOS patients, directly and/or indirectly promotes androgen synthesis and secretion (41, 42). Subsequent hyperandrogenism stimulates visceral adipose tissue lipolysis, increasing free fatty acids that exacerbate IR (42). Concurrently, IR aggravates hormonal dysregulation, inflammatory responses, and visceral adiposity—key drivers of obesity (43). These interconnected abnormalities establish a vicious cycle, worsening infertility, acne, and hirsutism, which profoundly impact self-image and amplify psychosocial stress (38, 44).

Acupuncture demonstrates potential in alleviating PCOS symptoms, with its antidepressant effects already validated (45, 46). This therapy physiologically regulates menstrual cycles, ovulation, and hyperandrogenism manifestations while mitigating adverse emotional impacts, all with minimal side effects (11, 47). Animal studies confirm that acupuncture reduces anxiety-like behaviors in female rats separated from their offspring by modulating the amygdala neuropeptide Y system (17). Qualitative research indicates that women with PCOS undergoing acupuncture treatment exhibit trends toward increased self-confidence, restored hope, and rebuilt autonomy (18). Through metabolic regulation, acupuncture modulates glucose and lipid metabolism to reduce obesity while enhancing fertility. Mechanistically, endocrine dysregulation in PCOS patients (e.g., insulin resistance, hyperandrogenism, chronic inflammation) induces anxiety and depression by altering neurotransmitters like dopamine and serotonin (48). Beyond regulating endocrine and metabolic markers, acupuncture’s potential mechanisms for alleviating anxiety and depression in PCOS patients may involve modulating multiple interconnected neurobiological pathways. First, acupuncture regulates the HPA axis—a core stress response system often hyperactive in mood disorders and PCOS. Preclinical studies indicate that electroacupuncture stimulation at specific points (e.g., ST36 Zusanli) reduces corticotropin-releasing hormone (CRH) expression and cortisol levels, thereby promoting HPA axis homeostasis and enhancing stress resilience (49, 50). Second, acupuncture influences monoaminergic neurotransmitter systems, particularly serotonin (5-HT) and norepinephrine systems. Evidence suggests acupuncture increases serotonin availability and modulates 5-HT1A receptor sensitivity in limbic brain regions (e.g., hippocampus, prefrontal cortex) critical for emotional regulation (15, 51). Furthermore, the insulin resistance and chronic low-grade inflammation prevalent in PCOS adversely affect neuroplasticity and neurotransmitter synthesis. Studies indicate that acupuncture improves insulin sensitivity and reduces levels of pro-inflammatory cytokines (e.g., TNF-*α*, IL-6), potentially creating a neurochemical environment conducive to emotional well-being (52, 53). Moreover, emerging research indicates that acupuncture may influence central nervous system function by regulating the gut-brain axis and modulating the production of gut microbiota metabolites (54–56). Collectively, acupuncture exerts holistic effects on emotional disorders through integrated physiological, metabolic, and phenotypic regulation.

### 4.2 Clinical significance and limitations

This study provides critical insights for the clinical application of acupuncture in managing anxiety and depression in polycystic ovary syndrome (PCOS) patients, establishing a theoretical foundation for standardized treatment protocols. Analysis of acupoints and meridians across the 12 included studies (Supplementary Table S2; Figure 2) revealed the most frequently used acupoints as Sanyinjiao (SP6), Guanyuan (CV4), Zusanli (ST36), Taichong (LR3), and Zigong (EX-CA1), with core meridians, including the Foot-Taiyin Spleen Meridian (SP), Conception Vessel (CV), Foot-Yangming Stomach Meridian (ST), and Foot-Jueyin Liver Meridian (LR). Traditional medicine emphasizes the regulatory roles of the Chong and Ren Meridians and the liver, spleen, and kidney organs in PCOS pathogenesis, demonstrating a mechanistic correspondence between the TCM “Kidney–Tian Gui–Chong Ren–Uterus” reproductive axis and the modern hypothalamic–pituitary–ovarian (HPO) axis (50). Through the synergistic effects of multiple targets and pathways mentioned above (57), the acupoint combinations summarized herein achieve holistic regulation of physiological, metabolic, and psychological dimensions in PCOS, circumventing the adverse effects of Western pharmaceuticals and the limitations of monotherapy while offering a novel integrative approach for emotional disorders.

Subgroup analyses (Supplementary Table S4) revealed the superior efficacy of manual acupuncture over electroacupuncture in alleviating anxiety/depression, with a shorter treatment duration (≤3 months) yielding more significant psychological improvement and larger effect sizes observed in smaller-sample studies (*n* ≤ 30). This heterogeneity primarily stems from nonstandardized acupuncture protocols (e.g., variable stimulation parameters) and methodological quality variations. Notably, while electroacupuncture efficacy fluctuates with parameter consistency (frequency, waveform), both modalities significantly outperform conventional treatments. Manual acupuncture employs personalized point selection (e.g., LR3, CV4, SP6) combined with lifting-thrusting-twisting techniques to elicit “Deqi” sensations (soreness, numbness, distension), stimulating deep vagal nerve fibers to modulate limbic system function (58). Its dual mechanisms of acupoint specificity and neuromodulation confer distinct advantages for emotional disorder intervention (58, 59). The marked efficacy of short-term therapy (≤3 months) in mild-to-moderate depression (60, 61) may reflect heightened neural sensitivity during acute phases—acupuncture rapidly alleviates symptoms by downregulating proinflammatory factors (e.g., TNF-*α*) and modulating the HPA axis (reducing ACTH and cortisol) (60).

This study has several limitations that may impact the reliability of the findings. (1) Few high-quality RCTs have specifically targeted acupuncture for improving anxiety and depression in patients with PCOS. Only 12 studies met the inclusion criteria, predominantly featuring small sample sizes (mostly 20–50 participants) and considerable size variation (range: 20–468 participants). (2) Effective blinding was difficult to implement due to the nature of the acupuncture interventions. Only three studies mentioned single−/double-blind designs. Overall study quality varied, introducing potential bias risk. (3) Acupuncture protocols vary significantly in terms of point selection and stimulation parameters. Most studies lacked standardized sham acupuncture controls, limiting the comparability of the results. (4) Most RCTs assessed outcomes only before and after treatment, and long-term follow-up data are lacking. This precludes the evaluation of treatment effects on sustainability and long-term mental health impacts. (5) Adverse effects have been underreported in some studies, and comprehensive safety evaluations are generally lacking. (6) The GRADE assessment of this study demonstrated that the evidence supporting acupuncture for improving anxiety and depression in patients with PCOS is of low quality, primarily due to the risk of bias in the primary studies and substantial heterogeneity among the results. Therefore, the conclusions drawn from this study require validation through future high-quality, large-scale RCTs incorporating long-term follow-up and standardized protocols.

## 5 Conclusion

This meta-analysis confirms that acupuncture (particularly manual acupuncture and short-term protocols) demonstrates superior efficacy over conventional treatments in alleviating emotional symptoms among patients with PCOS. In alignment with the emphasis on nonpharmacological therapies in the 2023 International Evidence-Based Guideline for PCOS (9), future clinical practice warrants exploration of an integrated acupuncture-psychology-lifestyle intervention model to improve patients’ physical and mental health outcomes holistically. Although acupuncture has promising therapeutic potential for treating PCOS, certain outcomes remain inconsistent, and its precise mechanisms have not yet been fully elucidated. Consequently, more rigorously designed, high-quality clinical studies are imperative to strengthen the credibility of these findings and further validate the long-term clinical efficacy of acupuncture in ameliorating anxiety and depression in patients with PCOS, thereby informing evidence-based clinical decision-making.

## Data Availability

The datasets presented in this study can be found in online repositories. The names of the repository/repositories and accession number(s) can be found in the article/Supplementary material.
