# Supplementary material for: Acupuncture improves anxiety and depression in patients with polycystic ovary syndrome: a systematic evaluation and meta-analysis
Source: Front Med (Lausanne). 2026 Jan 21;13:1738629. doi: 10.3389/fmed.2026.1738629 (PMC12868136; doi:10.3389/fmed.2026.1738629)
Supplement: Supplementary file 1 [file Supplementary_file_1.docx]

**Table S1.** Search Strategies

**Pubmed：**

((("Polycystic Ovary Syndrome"[Mesh]) OR ((((((((((((((Ovary Syndrome, Polycystic) OR (Syndrome, Polycystic Ovary)) OR (Stein-Leventhal Syndrome)) OR (Stein Leventhal Syndrome)) OR (Syndrome, Stein-Leventhal)) OR (Sclerocystic Ovarian Degeneration)) OR (Ovarian Degeneration, Sclerocystic)) OR (Sclerocystic Ovary Syndrome)) OR (Polycystic Ovarian Syndrome)) OR (Ovarian Syndrome, Polycystic)) OR (Polycystic Ovary Syndrome 1)) OR (Sclerocystic Ovaries)) OR (Ovary, Sclerocystic)) OR (Sclerocystic Ovary)))

AND

(((("Acupuncture"[MeSH]) OR (Pharmacopuncture)) OR (Electroacupuncture)) OR (Manual Acupuncture)) OR (Ear acupuncture)

AND

((("Randomized Controlled Trial"[MeSH]) OR (Clinical Trial, Randomized)) OR (Trial, Randomized Clinical)) OR (Controlled Clinical trial, Randomized)

**Embase:**

(exp polycystic ovary syndrome/ OR "polycystic ovary syndrome".ti,ab OR "polycystic ovarian syndrome".ti,ab OR "polycystic ovary disease".ti,ab OR "Stein-Leventhal Syndrome".ti,ab OR PCOS.ti,ab)

AND

(exp acupuncture/ OR exp moxibustion/ OR acupuncture.ti,ab OR electroacupuncture.ti,ab OR manual acupuncture.ti,ab OR "ear acupuncture".ti,ab)

AND

(exp randomized controlled trial/ OR "randomized controlled trial".ti,ab OR "randomised controlled trial".ti,ab OR randomied controlled trial.ti,ab OR RCT.ti,ab )

**Web of Science:**

TS = (("Polycystic Ovary Syndrome" OR "Polycystic Ovarian Syndrome" OR "Polycystic Ovary Disease" OR "Stein-Leventhal Syndrome" OR PCOS)

AND

(acupuncture OR electroacupuncture OR manual acupuncture OR "ear acupuncture" )

AND

("randomized controlled trial" OR "randomised controlled trial" OR RCT)

**CNKI/Wanfang/VIP/Duxiu:**

(Polycystic Ovary Syndrome OR Polycystic Ovary Syndrome OR PCOS)

AND

(Acupuncture OR electroacupuncture OR manual acupuncture OR acupoint acupuncture OR ear acupuncture)

AND

(randomized controlled trial OR randomized controlled study OR RCT)

**Table S2.** Analysis of Meridian and Acupoint Application Patterns in Acupuncture for PCOS-Related Anxiety and Depression Based on Data Mining

**Footnote:** Data were mined from 12 included RCTs (n=9 studies reporting specific acupoints). Frequency indicates the number of studies in which the meridian or acupoint was used. The combinations represent the most frequently co-occurring acupoint pairs or groups within the analyzed acupuncture prescriptions.

| **Category** | **Meridian / Acupoint** | **Frequency (n)** | **Frequency (%)** | **Exemplary Key Combinations (Frequency)** |
| --- | --- | --- | --- | --- |
| **High-Frequency Meridians** | Spleen Meridian (SP) | 9 | 100% | SP6 (9), SP10 (1), SP9 (1) |
|  | Conception Vessel (CV) | 8 | 89% | CV4 (8), CV3 (5), CV6 (3), CV12 (3) |
|  | Stomach Meridian (ST) | 8 | 89% | ST36 (8), ST25 (4), ST40 (3) |
|  | Liver Meridian (LR) | 7 | 78% | LR3 (6), LR2 (1), LR14 (1) |
| **Core Acupoints** | Sanyinjiao (SP6) | 9 | 100% | CV4 + SP6 (8), ST36 + SP6 (7), LR3 + SP6 (6) |
|  | Guanyuan (CV4) | 8 | 89% | CV4 + ST36 + SP6 (7), GV20 + CV4 (5) |
|  | Zusanli (ST36) | 8 | 89% | ST36 + SP6 (7), ST25 + ST36 (4) |
|  | Taichong (LR3) | 6 | 67% | LR3 + SP6 (6) |
|  | Zigong (EX-CA1) | 5 | 56% | CV4 + EX-CA1 (4) |
|  | Baihui (GV20) | 5 | 56% | GV20 + CV4 (5) |
| **Associated Meridians** | Governor Vessel (GV) | 5 | 56% | GV20 (5) |
|  | Bladder Meridian (BL) | 4 | 44% | BL18 (3), BL20 (3), BL23 (2) |
|  | Large Intestine Meridian (LI) | 3 | 33% | LI4 (3) |

**Table S3.** Assessment according to the GRADE system.

| **Outcome** | **No. of Studies (Participants)** | **Risk of Bias** | **Inconsistency** | **Imprecision** | **Publication Bias** | **Quality of Evidence** | **Importance** |
| --- | --- | --- | --- | --- | --- | --- | --- |
| **Anxiety State (SAS)** | 12 (n=2127) | Serious | Serious | Not Serious | Likely | LOW | Important |
| **Depression State (SDS)** | 10 (n=2021) | Serious | Serious | Not Serious | Likely | LOW | Important |
| **Testosterone (T) Level** | 5 (n=785) | Serious | Not Serious | Not Serious | Potential | MODERATE | Critical |
| **Insulin Resistance (HOMA-IR)** | 5 (n=818) | Serious | Serious | Serious | Likely | VERY LOW | Critical |
| **Body Mass Index (BMI)** | 7 (n=1138) | Serious | Serious | Not Serious | Potential | LOW | Important |
| **Waist-Hip Ratio (WHR)** | 4 (n=698) | Serious | Serious | Serious | Potential | VERY LOW | Critical |
| **Adverse Events** | 4 (n=365) | Serious | Serious | Serious | Potential | LOW | Important |

**Table S4.** Forest Plot of Subgroup Analysis Results for Outcome Measures

| **Outcome or Subgroup** | **Results of the Meta-Analysis** | | | |
| --- | --- | --- | --- | --- |
|  | **No of studies** | **No of sample** | **Heterogeneity** | **Mean Difference (IV, Random, 95% CI)** |
| **1.SAS** | 12 | 2113 | I2=98%,P<0.00001 | -6.24 [-8.91, -3.56] |
| 1.1 Intervention measure |  |  |  |  |
| EA | 6 | 1733 | I2=90%,P<0.00001 | -3.55 [-6.47, -0.62] |
| MA | 6 | 380 | I2=96%,P<0.00001 | -8.78 [-11.62, -5.94] |
| 1.2 Treatment course |  |  |  |  |
| ≤3 months | 6 | 558 | I2=95%,P<0.00001 | -7.04 [-9.28, -4.80] |
| ＞3 months | 6 | 1565 | I2=91%,P<0.00001 | -2.78 [-5.72, 0.16] |
| 1.3 Sample size |  |  |  |  |
| n≤30 | 6 | 323 | I2=95%,P<0.00001 | -7.44 [-10.48, -4.41] |
| n>30 | 6 | 1750 | I2=97%,P<0.00001 | -4.95 [-9.81, -0.09] |
| **2.SDS** | 10 | 1953 | I2=98%,P<0.00001 | -5.89 [-9.01, -2.78] |
| 2.1 Intervention measure |  |  |  |  |
| EA | 4 | 1573 | I2=84%,P=0.0004 | -2.72 [-5.41, -0.02] |
| MA | 6 | 380 | I2=92%,P<0.00001 | -7.95 [-10.56, -5.33] |
| 2.2 Treatment course |  |  |  |  |
| ≤3 months | 7 | 488 | I2=91%,P<0.00001 | -7.65 [-10.01, -5.30] |
| ＞3 months | 3 | 1465 | I2=69%,P=0.04 | -1.38 [-3.55, 0.79] |
| 2.3 Sample size |  |  |  |  |
| n≤30 | 6 | 330 | I2=88%,P<0.00001 | -6.63 [-9.07, -4.20] |
| n>30 | 4 | 1623 | I2=98%,P<0.00001 | -5.20 [-11.35, 0.96] |
| **3.Total testosteroneMean(T)** | 5 | 749 | I2=0%,P=0.47 | -0.05 [-0.11, 0.00] |
| **4.HOMA-IR** | 5 | 1666 | I2=99%,P<0.00001 | -0.73 [-2.53, 1.07] |
| 4.1 Intervention measure |  |  |  |  |
| EA | 4 | 1606 | I2=99%,P<0.00001 | -0.61 [-2.75, 1.52] |
| MA | 1 | 60 | NA | -1.16 [-1.92, -0.40] |
| 4.2 Treatment course |  |  |  |  |
| ≤3 months | 2 | 168 | I2=0%,P=0.90 | -1.11 [-1.20, -1.02] |
| ＞3 months | 3 | 1498 | I2=99%,P<0.00001 | -1.11 [-5.64, 3.42] |
| 4.3 Sample size |  |  |  |  |
| n≤30 | 1 | 60 | NA | -1.16 [-1.92, -0.40] |
| n>30 | 4 | 1606 | I2=99%,P<0.00001 | -0.61 [-2.75, 1.52] |
| **5.Body Mass Index(BMI)** | 7 | 1789 | I2=60%,P=0.02 | -0.68 [-0.97, -0.40] |
| 5.1 Intervention measure |  |  |  |  |
| EA | 5 | 1669 | I2=58%,P=0.05 | -0.85 [-1.17, -0.54] |
| MA | 2 | 120 | I2=0%,P=0.79 | -0.03 [-0.65, 0.59] |
| 5.2 Treatment course |  |  |  |  |
| ≤3 months | 3 | 228 | I2=85%,P=0.001 | -0.85 [-1.30, -0.41] |
| ＞3 months | 4 | 1561 | I2=0%,P=0.97 | -0.57 [-0.93, -0.20] |
| 5.3 Sample size |  |  |  |  |
| n≤30 | 3 | 160 | I2=0%,P=0.55 | -0.17 [-0.74, 0.40] |
| n>30 | 4 | 1629 | I2=69%,P=0.02 | -0.85 [-1.18, -0.53] |
| **6.Waist to hip ratio(WHR)** | 4 | 1629 | I2=93%,P<0.00001 | 0.06(-0.11,-0.01) |
| 6.1 Treatment course |  |  |  |  |
| ≤3 months | 1 | 108 | NA | -0.08 [-0.12, -0.04] |
| ＞3 months | 3 | 1521 | I2=95%,P<0.00001 | -0.09 [-0.19, -0.01] |

**Footnote:** SAS: Self-Rating Anxiety Scale score; SDS: Self-Rating Depression Scale score; T: Testosterone; HOMA-IR: Homeostatic model assessment of insulin resistance; BMI: Body mass index; WHR: Waist-to-hip ratio; NA: Not applicable; I²: Heterogeneity (I-squared statistic)

**Table S5.** Egger's test

| **Name** | **t** | **P > \|t\|** | **95% CI** | |
| --- | --- | --- | --- | --- |
| **SAS** | 0.79 | 0.445 | -3.352075 | 7.070816 |
| **SDS** | 1.50 | 0.172 | -1.620785 | 7.652352 |
